# Effects of mental fatigue on the psychophysiological responses, kinematic profiles, and technical performance in different small-sided soccer games

**DOI:** 10.5114/biolsport.2022.110746

**Published:** 2021-12-30

**Authors:** Yusuf Soylu, Fikret Ramazanoglu, Ersan Arslan, Filipe Manuel Clemente

**Affiliations:** 1 Tokat Gaziosmanpasa University, Faculty of Sport Sciences, Tokat, Turkey; 2 Sakarya University of Applied Sciences, Faculty of Sport Sciences, Sakarya, Turkey; 3 Escola Superior Desporto e Lazer, Instituto Politécnico de Viana do Castelo, Rua Escola Industrial e Comercial de Nun’Álvares, 4900-347, Viana do Castelo, Portugal

**Keywords:** Game-based training, Perceived exertion, Physical, Enjoyment, Psychological responses, Mental, effort, Technical activities

## Abstract

The purpose of this study was to assess the effects of mental fatigue (MF) on the psychophysiological responses, kinematic profiles, and technical performance of young soccer players in small-sided games (SSGs). Twenty-four young soccer players (age: 15.9 ± 1.0 years) played 2vs2, 3vs3, and 4vs4 SSGs consisting of four bouts (with two-minute passive rest periods between bouts) under two different playing conditions: MF+SSGs and SSGs. The heart rate, total distance covered, and technical performance of each player were monitored during all SSGs, and the rating of perceived exertion, visual analogue scale, and Rating Scale Mental Effort values were determined after each bout. The Physical Activity Enjoyment Scale (PACES) and Brunel Mood Scale (BRUMS) were also determined at the end of each SSG. The results demonstrated that all MF+SSGs induced higher psychophysiological responses (p ≤ 0.05) than SSGs, except regarding the PACES responses. By contrast, the SSGs group covered a greater total distance (p ≤ 0.05) than the MF+SSGs group. During SSGs, the players’ technical performances (in terms of lost balls and unsuccessful passes) were negatively affected after MF (p ≤ 0.05). The results of this study indicate that both PACES scores and mood responses were negatively affected after the MF intervention. Coaches could use the MF intervention before SSGs to improve soccer-specific technical and decision-making performances in young soccer players.

## INTRODUCTION

Soccer is characterized by a combination of physical performances, including running at different speeds, physical challenges, and actions requiring technical skills with balls (e.g., shooting, dribbling, and tackling). Many studies have shown that young players perform at an average game intensity ranging from 80% to 86% of their maximum heart rate during soccer matches [1, 2]. Furthermore, recent studies have shown that young soccer players (16–18 years old) cover distances between 8.5 and 9.9 km and that high-intensity activity (13–18 km · h^-1^) accounts for approximately 8.6–12% of the total distance covered during a competitive soccer match [3, 4]. Consequently, these performance results, including superior aerobic endurance and anaerobic capacity, are required for players to maintain high levels of performance and perform repeated high-intensity intermittent efforts during official matches [5, 6]. Therefore, many sports scientists and coaches have recently focused on improving aerobic fitness with technical and tactical stimuli using small-sided games (SSGs) in young soccer players.

In contrast to the traditional aerobic strategy, SSGs provide an enjoyable, effective, and time-efficient training method. These games simultaneously involve actual movement patterns, technical-tactical awareness, and physical fitness under simulated game conditions [7–9]. Numerous studies have reported that various factors, such as coach encouragement [10], resting regime [11], and training regime [7], affect players’ performances during SSGs. In addition to these factors, the number of players [8], pitch size [12], and rule modifications [13] could affect the intensity and demands of SSGs. Recently, one additional popular psychobiological factor – mental fatigue (MF) – has been characterized by increased feelings of tiredness and a lack of energy [14, 15], as well as decreased performance related to specific game-based technical such as shooting, passing, and controlling the ball [16]. Consequently, this situation may not only change the game performance qualitatively but also influence the speed and accuracy of shots quantitatively in young soccer players during the SSGs.

SSGs might provide a valid ecological condition to assess the effect of MF on the performance of players under simulated game conditions. In the last five years, an increasing number of studies have confirmed the adverse effects of MF on soccer-specific physical [18–20], technical/tactical [17, 21, 22], and decision-making [16, 22] performance among players of different ages and skill levels during SSGs. For example, Badin et al. [17] demonstrated that MF impaired the technical performance of young soccer players during 5vs5 SSGs. In another similar recent study, Trecroci et al. [22] investigated potential MF-related impairments of physical activity and technical and decision-making performance during 4vs4 (plus one wildcard player) SSGs. The authors found that SSG performance was negatively affected by MF after a 30-min Stroop colour-word task in young sub-elite soccer players.

Numerous recent studies have compared the effects of variety in MF interventions on the tactical, technical, and decision-making performance of players during SSGs [21, 23, 24]. However, no studies have investigated the effects of MF interventions on the psychophysiological responses, time-motion characteristics, and technical performances in soccer players in a single study. To the best of our knowledge, this study is the first to examine these variables in detail in youth soccer players. Therefore, the purpose of this study was to investigate the effects of MF intervention on the psychophysiological responses, time-motion characteristics, and technical performances in young soccer players. We hypothesized that the MF intervention would worsen players’ game-based relevant performance responses such as physical enjoyment, total distance covered, and technical actions (e.g., lost balls and unsuccessful passes).

## MATERIALS AND METHODS

### Experimental Approach to the Problem

A counterbalanced design was used to assess the effects of MF on the psychophysiological responses, kinematic profiles, and technical performance of young soccer players in different SSGs. Players were assigned to two groups – an MF+SSGs group and an SSGs group – according to the distance covered in the Yo-Yo Intermittent Recovery Test Level-1 (YYIRTL-1). Three MF intervention sessions were carried out in the morning during the pre-season training period. To avoid potential negative influences of MF interventions on performance in SSGs, MF intervention sessions were separated by at least seven days [20, 23]. In addition, SSGs were separated by at least three days to avoid any possible negative effects of physical and physiological fatigue. Small goals were used in all SSGs to simulate physiological game characteristics with offensive and defensive tactics on the pitch. After MF interventions, all SSGs were performed on a natural grass pitch at a similar time of the day to ensure that the chronobiological characteristics were similar across trials [25, 26]. Players were advised not to perform moderate- to high-intensity exercise within 24 hours before the MF intervention; they were also instructed to get at least 7–8 hours of sleep. The players were also advised not to drink alcoholic or caffeinated beverages within 24 hours prior to the MF intervention. The indoor and outdoor temperatures (25–28°C) and humidity (35–40%) were similar during the study.

### Subjects

Twenty-four young male soccer players (age: 15.9 ± 1.0 years, body height 172.1 ± 7.2 cm, body mass 58.7 ± 8.4 kg; body fat % 13.7 ± 3.6) participated in this study during the 2018–2019 preseason period. All the players were members of the same young soccer team, competing in a U-16 regional development league. The players were familiar with a training workload of > 4 training units per week and they have been involved in soccer training and league matches for more than 2 years. Before signing the informed consent form, players and their parents were notified of the research benefits, requirements, procedures and potential risks. Then they all provided written consent for participation. The present study was approved by the Research Ethics Committee (26428519/100/), and was conducted in accordance with the Declaration of Helsinki.

### Procedures

**FIG. 1:**
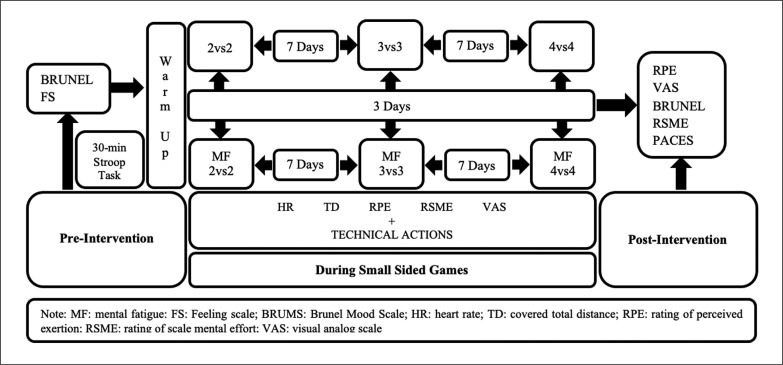
Study design.

Testing Sessions. A total of seven sessions (one familiarization session and six testing sessions) (MF+SSGs and SSGs) were completed during the present study. The players were familiar with all testing procedures before the study began. Each participant wore an heart rate (HR) monitor and responded to psychophysiological scales such as rating of perceived exertion (RPE), the Physical Activity Enjoyment Scale (PACES), and the Rating Scale Mental Effort (RSME) during their daily training routines for at least two years. Before the YYIRTL-1, participants were given all required information about the experimental protocol and underwent a familiarization session to understand the testing procedures. Specifically, players were fully informed about the MF intervention. In order to assess players’ aerobic fitness, the YY-IRTL-1, which is a reliable and acoustically popular progressive field test [26], was performed on a natural grass pitch according to procedures suggested by Bangsbo et al. [27]. After the test, the players were ranked based on their aerobic fitness level, from highest to lowest, according to the distance covered in the YYIRTL-1 to avoid having SSG teams unbalanced in aerobic fitness level. The intervention procedure is summarized in Figure 1. When all players arrived at the soccer club facility, they were given a left wrist-worn triaxial accelerometer (50 Hz) based model HR monitor (Polar M430, Kempele, Finland) to measure their HR (1-second interval) and the total distance covered during all sessions. Henriksen et al. [28] found that the Polar M430 demonstrated a moderate to strong relationship (r = 0.59–0.76) with accelerometer-based tools such as the ActiGraph and Actiheart for total distance covered measured for different intensity physical activities (ranging from moderate to very vigorous). At the same time, their results on the Feeling Scale (FS) and Brunel Mood Scale (BRUMS) were assessed in order to determine their physical fatigue and mood profile, respectively, before and after the MF intervention. For the FS, the 11-point bipolar measurement scale – with scores ranging from -5 (very bad) to +5 (very good) – was used to assess affective valence [29]. Terry et al. [30] developed the BRUMS and included 19 items scored (e.g., angry, energetic, nervous, or unhappy), on a five-point Likert scale (0 = not at all, 1 = a little, 2 = moderately, 3 = quite a bit, 4 = extremely) using the “How do you feel right now?” response timeframe. This scale was determined to be valid and reliable for evaluating Turkish athletes’ mood profiles [31].

**FIG. 2:**
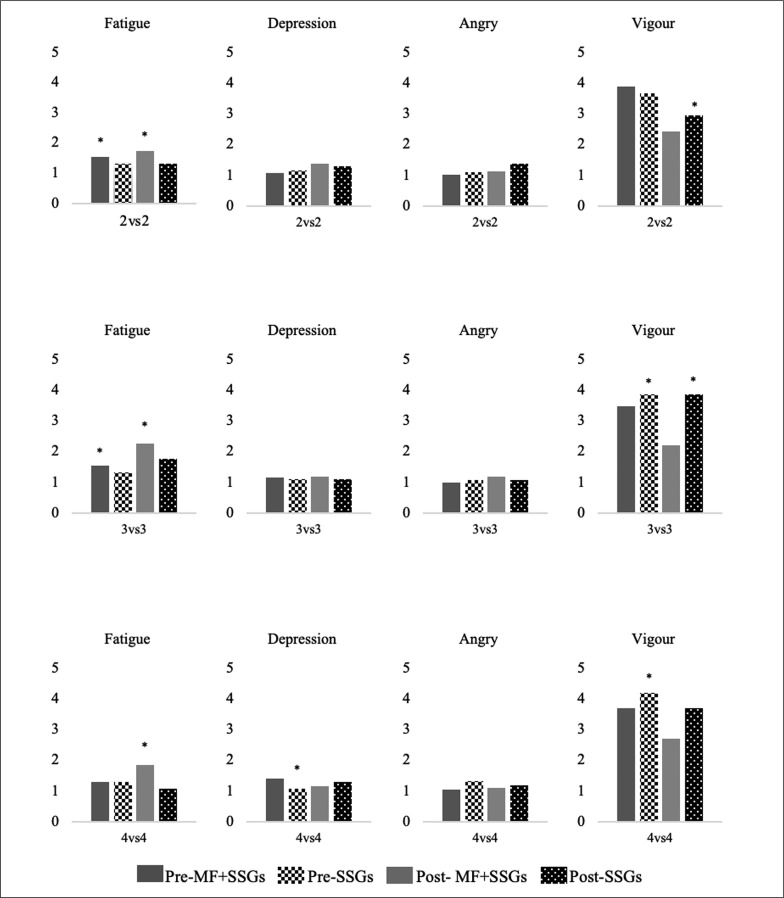
Mood responses.

Mental Fatigue Intervention and SSGs. All players were divided into three groups to complete a 30-min paper coloured version of the Stroop task in quiet rooms according to the same protocol as used in similar previous studies [21, 22]. It was previously shown that this prolonged cognitive task requires intensive attention and automatic response inhibition, and induces mental fatigue due to the cognitive manipulations [32]. The Stroop task, consisting of four words (red, blue, green and yellow), was shown in a random order, following by a 1.5 s resting interval. Players were also instructed to quickly and accurately finish the task in competition with the other teammates. Following the MF intervention, all players performed a 15-min standardized warm-up, consisting of jogging and dynamic stretching with integration of soccer-specific actions. The detailed features of SSGs are illustrated in Table 1. HR and covered total distance were continuously measured during all the SSGs. The RPE (assessing for physical fatigue) and 100-mm visual analogue scale (VAS, assessing for MF fatigue) were also reported by players after each bout of SSGs. These highly validated and reliable scales were frequently used to measure the level of physical and MF according to previous studies [17, 22, 33]. Furthermore, the RSME was used to assess the subjective mental effort during all the SSGs. The scale, which has a good relationship with performance [34], is a single item to assess to mental workload ranging from no effort (0) to extreme effort (150) [35]. Following the SSGs, BRUMS and PACES scores were used to measure players’ mood (Figure 2) and enjoyment responses. After 10 min of all SSGs, players answered the short form of the PACES. This is a scale for physical enjoyment level, including 5 items scored on a 1–7 Likert scale, validated in Turkish youths [36]. During the SSGs, technical performances of young soccer players were recorded using a high-definition video camera (Canon LEGRIA HF R806, Tokyo, Japan) and analysed with a specialised soccer analysis program, eAnalyze Soccer (Espor Digital, Ankara, Turkey). The important technical actions, including pass (successful and unsuccessful), interception, lost ball, shot and goal, were selected for analysis and the technical analysis was performed by an experienced soccer coach with a UEFA B license.

**TABLE 1:** The features of all small-sided games

	2-a-side		3-a-side		4-a-side	
	MF+SSGs	SSGs	MF+SSGs	SSGs	MF+SSGs	SSGs
Number of Bouts	4		4		4	
Bout Duration (min)	2		3		4	
Resting Duration (min)	2		2		2	
Pitch Dimension (mxm)	15 × 27		20 × 30		25 × 32	
Relative Pitch Size (m^2^)	1:100					
Small goals	Yes					

MF: mental fatigue; SSGs: small-sided games.

### Statistical Analyses

Data were represented as mean ± SD. A paired t-test was performed on each dependent variable, including psychophysiological responses, kinematic profile, and technical performances, in order to compare differences between the MF+SSGs and SSGs conditions for all games. Inter-individual variability in psychophysiological responses, kinematic profile, and technical performances between the MF+SSGs and SSGs conditions was quantified using the coefficient of variation (CV). Effect sizes (Cohen’s *d*) were also calculated for each dependent variable. The thresholds for effect size statistics were as follows: 0.2, trivial; 0.6, small; 1.2, moderate; 2.0, large; and .2.0, very large [37]. Statistical analyses were performed with SPSS software version 24.0 (SPSS Inc, Chicago, IL, USA). The level of statistical significance was set at p ≤ 0.05.

## RESULTS

Table 2 and Figure 1 demonstrate the psychophysiological responses and kinematic profiles of youth soccer players during the 2vs2, 3vs3, and 4vs4 SSGs under MF+SSGs and SSGs conditions. All MF+SSGs formats induced significantly higher psychophysiological responses in terms of HR, %HR_max_, RPE, VAS and RSME responses compared with SSGs conditions (*p* ≤ 0.05, *d* = ranging from 0.43 to 1.12 [small to moderate effect]). Conversely, PACES responses in 2vs2, 3vs3, and 4vs4 SSGs were significantly higher than those in 2vs2 (t = 2.208; *p* = 0.37; *d* = 0.36 [small effect]; 3vs3 (t = 2.087; *p* = 0.48; *d* = 0.39 [small effect]; and 4vs4 (t = 2.084; *p* = 0.48; *d* = 0.51 [small effect] formats of MF+SSGs. Moreover, total distances covered in 2vs2, 3vs3, and 4vs4 SSGs were significantly higher than those in 2vs2 (t = 3.862; *p* = 0.00; *d* = 1.08 [moderate effect]; 3vs3 (t = 3.010; *p* = 0.48; *d* = 0.00 [moderate effect]; and 4vs4 (t = 2.281; *p* = 0.03; *d* = 0.48 [small effect] formats of MF+SSGs.

**TABLE 2:** Psychophysiological responses and kinematic profiles of young soccer players

			MF+SSGs	CV %	SSGs	CV %	Mean Difference	%95CI Lower – Upper	*d*	Magnitude
Psychophysiological responses and kinematic profiles	2vs2	Total Distance (m)	1024.7 ± 134.3	13.11	1141.1 ± 73.1*	6.40	-116.4	54.05 to 178.70	1.08	Moderate
		HR (beat · min^-1^)	178.3 ± 3.6*	1.97	174.2 ± 4.2	2.39	4.1	-6.35 to -1.89	1.05	Moderate
		%HR_max_	90.0 ± 1.7*	1.82	87.9 ± 2.9	3.27	2.1	-3.23 to -0.92	0.88	Moderate
		RPE	15.9 ± 1.4*	9.33	15.2 ± 1.4	10.82	0.6	0.23 to -0.10	0.50	Small
		VAS	4.8 ± 1.9*	50.13	3.8 ± 1.9	57.14	1.0	-1.78 to -0.21	0.53	Small
		RSME	100.8 ± 21.2*	21.03	90.1 ± 27.9	30.96	10.7	-20.86 to -0.55	0.43	Small
		PACES	26.9 ± 6.2	24.91	28.8 ± 4.0*	14.20	-1.9	0.12 to -3.71	0.36	Small
	3vs3	Total Distance (m)	1500.3 ± 116.0	7.73	1620.2 ± 130*	8.25	-119.9	37.50 to 202.33	0.96	Moderate
		HR (beat · min^-1^)	180.6 ± 6.4*	3.53	174.5 ± 4.8	2.73	6.1	-8.89 to -3.35	1.08	Moderate
		%HR_max_	91.2 ± 3.0*	3.34	88.0 ± 2.7	3.11	3.2	-4.47 to -1.81	1.12	Moderate
		RPE	16.5 ± 2.6*	14.95	14.5 ± 1.4	12.95	2.0	-2.91 to -1.08	0.96	Moderate
		VAS	5.7 ± 2.5*	53.48	4.3 ± 1.9	44.31	1.4	-4.24 to -2.50	0.63	Moderate
		RSME	97.3 ± 26.2*	26.92	85.8 ± 23.4	27.27	11.5	-21.37 to -1.54	0.46	Small
		PACES	26.3 ± 7.3	31.72	28.7 ± 4.9*	17.95	-2.4	0.02 to 4.81	0.39	Small
	4vs4	Total Distance (m)	1994.7 ± 325.7	11.82	2122.9 ± 271*	13.03	-128.2	11.95 to 244.38	0.42	Small
		HR (beat · min^-1^)	176.6 ± 6.8*	3.82	172.0 ± 7.5	4.38	4.6	-8.08 to -1.07	0.64	Moderate
		%HR_max_	89.2 ± 3.8*	4.25	86.9 ± 3.7	4.24	2.3	-4.03 to -0.57	0.61	Moderate
		RPE	15.7 ± 2.3*	13.57	14.3 ± 1.4	13.70	1.4	-2.24 to -0.59	0.96	Moderate
		VAS	4.6 ± 1.9*	49.61	3.5 ± 1.7	39.87	1.1	-1.88 to -0.20	0.61	Moderate
		RSME	91.9 ± 26.2*	28.50	77.9 ± 28.9	37.09	14	-22.56 to -5.35	0.51	Small
		PACES	26.5 ± 4.7	18.33	29.0 ± 5.0*	17.98	-2.5	0.01 to 4.89	0.51	Small

Data are Mean ± SD. MF+SSGs: small-sided games after mental fatigue; SSGs: small-sided games; HR: heart rate; %HR_max_: percentage of maximum heart rate; RPE: rating of perceived exertion; VAS: visual analog scale; RSME: rating of scale mental effort; PACES: physical activity enjoyment scale; CV: coefficient of variation; %95CI: 95% confidence interval (95% CI was estimated for the difference between two means; *d*: effect size (absolute value);

* Significant difference p ≤ 0.05.

Table 3 shows the technical activities of young soccer players during the 2vs2, 3vs3, and 4vs4 SSGs under MF+SSGs and SSGs conditions.

**TABLE 3:** Technical responses of young soccer players during the all SSGs

			MF+SSG	CV %	SSG	CV %	Mean Difference	%95CI Lower – Upper	*d*	Magnitude
Technical responses	2vs2	Succ Pass	14.15 ± 4.16	29.39	15.25 ± 4.74	34.21	-1.1	-2.61 to -4.81	0.25	Small
		UnSucc Pass	2.55 ± 0.51	20.02	2.85 ± 1.46	48.53	-0.3	-0.44 to -1.04	0.27	Small
		Interception	1.00 ± 0.00	0.00	1.59 ± 0.50*	28.89	-0.5	0.32 to 0.84	1.67	Large
		Lost Ball	3.42 ± 0.83*	24.28	2.12 ± 0.34	15.90	1.3	-1.65 to -0.92	2.05	Very Large
		Succ Shot	1.58 ± 0.50	32.40	3.17 ± 1.00*	31.81	-1.5	0.96 to 1.95	2.01	Very Large
		UnSucc Shot	3.16 ± 1.00	31.74	2.75 ± 1.42	51.70	0.4	-1.02 to 0.19	0.33	Small
		Goal	1.79 ± 0.41	23.15	3.67 ± 0.76*	20.77	-1.8	1.51 to 2.23	3.08	Very Large
	3vs3	Succ Pass	19.58 ± 9.54	48.70	21.71 ± 8.90	40.99	-2.1	-3.26 to 7.51	0.23	Small
		UnSucc Pass	4.79 ± 2.21*	46.04	3.62 ± 0.49	13.64	1.1	-3.60 to -1.39	0.72	Moderate
		Interception	0.75 ± 0.44	58.98	1.04 ± 0.20*	19.60	-0.2	0.05 to 0.52	0.84	Moderate
		Lost Ball	1.83 ± 0.38	20.77	1.75 ± 0.44	25.28	0.08	1.19 to 1.97	0.19	Trivial
		Succ Shot	2.50 ± 0.72	28.89	2.17 ± 0.38	17.57	0.3	-1.12 to -0.46	0.57	Small
		UnSucc Shot	2.33 ± 0.48	20.64	2.25 ± 0.44	19.66	0.08	-0.32 to 0.16	0.17	Trivial
		Goal	2.37 ± 0.74	27.24	2.10 ± 0.54	25.26	0.2	-0.58 to 0.08	0.42	Small
	4vs4	Succ Pass	19.67 ± 6.50	33.08	26.79 ± 7.17*	26.76	-7.12	2.82 to 11.42	1.04	Moderate
		UnSucc Pass	4.83 ± 1.00*	20.84	4.08 ± 0.28	6.91	0.7	-1.14 to -0.35	1.02	Moderate
		Interception	1.00 ± 0.00	0.00	1.06 ± 0.26	26.06	-0.06	-0.07 to 0.20	0.33	Small
		Lost Ball	2.00 ± 0.00*	0.00	1.21 ± 0.41	34.33	0.7	-0.96 to -0.61	2.72	Very Large
		Succ Shot	1.58 ± 0.50	31.81	1.96 ± 0.20*	10.42	-0.3	0.13 to 0.61	1.00	Moderate
		UnSucc Shot	1.83 ± 0.38	20.77	1.75 ± 0.44	25.28	0.08	-0.32 to 0.16	0.19	Trivial
		Goal	1.78 ± 0.42	23.66	1.61 ± 0.50	30.43	0.01	-0.48 to 0.13	0.37	Small

Data are Mean ± SD. MF+SSG: small-sided games after mental fatigue; SSG: small-sided games; Succ Pass: successful pass; UnSucc Pass: unsuccessful pass; CV: coefficient of variation; %95CI: 95% confidence interval (95% CI was estimated for the difference between two means; *d*: effect size (absolute value);

* Significant difference p ≤ 0.05.

## DISCUSSION

The purpose of this study was to assess the effects of MF on the psychophysiological responses, kinematic profiles, and technical performance of young soccer players in SSGs. The current research revealed that induced MF significantly and moderately increased the internal load measures of HR and RPE while significantly decreasing the total distance covered irrespectively of the MF+SSGs formats tested. Increases of significant but small magnitudes were also found in mental effort after the MF induced condition, while enjoyment was significantly better in formats without the MF condition. The impact on technical performance was mainly associated with passes, interceptions, and shots.

Regarding the effects of the MF condition on psychophysiological measures (i.e., HR, RPE, VAS), a significant and meaningful increase in MF led to increased intensities of the measures reported. In fact, independently of the formats of play, significant increases in HR_mean_, HR_max_, RPE, and VAS were found in the SSGs played after the MF intervention. These findings are consistent with previous research [17, 33]. In a study conducted in under-18 players to test the effects of MF in RPE reported after the 5vs5 format, it was found that subjective ratings of effort were significantly higher following the Stroop task [17]. Similar findings were reported in under-16 players in 6vs6 SSGs [33]. Such evidence might be related to changes in behaviour after inducing MF, which may have detrimental effects on endurance performance [38]. Therefore, it must be considered that MF can contribute significantly to effort perception during SSGs and increase the impact of internal load on players. It is expected that coaches will track such consequences to manage the load in accordance with the mental status of players.

Regarding the impact of MF on the kinematic profile during SSGs, there were significant decreases in total running distance covered in all the formats applied in the current study. In contrast to our findings, no significant difference in total distance between MF and neutral conditions was found in similar previous studies that researched the same topic in SSGs [19, 22, 33]. Only one study revealed a significant detrimental effect on distance covered [21]. Some explanations for this might be related to the reduced time of exercise (SSGs), as some evidence suggests that the shorter and more maximal the task, the smaller is the impact of MF [38]. In fact, total distance covered seems to be highly influenced not only by MF but also by high physical demands (e.g., high-intensity running and high-intensity accelerations or decelerations) [19, 22]. Therefore, MF does not seem to play a determinant role in influencing the external load during SSGs. Despite that, it is possible that longer SSGs (continuous regimen) suffer from the MF effect since endurance performance seems to be strongly influenced by MF due to the increased perceived effort [38].

In conjunction with MF’s adverse effect on kinematic profile, the current research found a significant and moderate detrimental impact of MF on the RSME. Such evidence reveals that the induced fatigue protocol had a clear impact on the mental effort of the players. Such a consequence should be considered by coaches since decision-making and tactical behaviour can be highly influenced by such constraints [19, 21]. In practical scenarios, MF might produce an influence that will change the decision-making of the players, thus likely changing the capacity of the players to reach the objective of the tasks in accordance with the coach’s intended purpose. The impact of MF also significantly affected in the present study the enjoyment of the players. In the neutral condition, enjoyment was meaningfully better. In fact, this finding can be related to the negative effect of adenosine on motivation and decreased commitment during the task [39]. Therefore, it is important that coaches control the negative effects of MF on the players’ enjoyment (and the potential links with motivation) while the players perform tasks.

Considering the impact of MF on technical performance, significant decreases were found in interceptions and shots. The results indicate that more interceptions and successful shots were made without MF. Additionally, MF significantly increased the number of unsuccessful passes. Considering previous findings, a great impact on successful passes is expected [22]. The present findings are in line with a previous study [22], thus confirming that MF may impair performance related to passing and other technical elements. This might be related to decision-making; therefore, future research should analyse the relationships between MF, mental effort, and affordances in soccer players. Furthermore, it is important that coaches consider which specific technical elements of players’ performance are negatively affected by MF.

The current study has some limitations. One of the main limitations is the small sample size, which could compromise the generalization of the evidence. Another limitation is that physical fitness was not considered as a covariable for the impact of MF on the players. A final limitation is the absence of data related to decision-making or tactical behaviour in the current study. Therefore, future research should consider increasing the sample size and determining the importance of covariables (such as expertise level or physical fitness) for the changes related to MF. Adding information about interaction between decision-making and tactical behaviours is also recommended.

## CONCLUSIONS

This study demonstrated that MF impacts soccer players’ psychophysiological responses, kinematic profiles, and technical performance in SSGs. This study also confirmed that MF is one of the psychobiological factors affecting the performance of players during SSGs. Thus, coaches and practitioners dealing with young players should consider MF prior to match days. SSGs, as time-efficient, effective, and enjoyable training strategies, might be preferable to reduce the adverse effects of MF. From a practical point of view, MF may have an adverse influence on the soccer-specific technical and decision-making performance of young soccer players. Therefore, it is important for coaches to control the negative effects of MF on players’ enjoyment and the potential links with motivation while performing tasks.

## Practical applications

Despite its limitations, this study presented consistent findings about the effects of MF on the training load, mental effort, and enjoyment of soccer players. In terms of practical implications, coaches should consider using instruments that allow them to monitor MF among players to reduce its potential impact on drill-based exercises conducted in the field. Finally, possible adjustments should be made to objectives or recovery strategies to mitigate the impact of MF on soccer players’ performance during SSGs.
